# Phase II study of tivantinib (ARQ 197) in patients with metastatic triple-negative breast cancer

**DOI:** 10.1007/s10637-015-0269-8

**Published:** 2015-07-01

**Authors:** Sara M. Tolaney, Sally Tan, Hao Guo, William Barry, Eliezer Van Allen, Nikhil Wagle, Jane Brock, Katherine Larrabee, Cloud Paweletz, Elena Ivanova, Pasi Janne, Beth Overmoyer, John J. Wright, Geoffrey I. Shapiro, Eric P. Winer, Ian E. Krop

**Affiliations:** Department of Medical Oncology, Dana-Farber Cancer Institute, 450 Brookline Avenue, Yawkey 1257, Boston, MA 02215 USA; Harvard Medical School, Boston, MA USA; Department of Biostatistics and Computation Biology, Dana-Farber Cancer Institute, Boston, MA USA; Cancer Program, Broad Institute of MIT and Harvard, Cambridge, MA USA; Department of Pathology, Brigham and Women’s Hospital, Boston, MA USA; Belfer Institute for Applied Cancer Sciences, Dana-Farber Cancer Institute, Boston, MA USA; Cancer Therapy Evaluation Program, National Cancer Institute, Bethesda, MD USA

**Keywords:** Triple-negative breast cancer, Tivantinib, MET, ARQ 197

## Abstract

*Background* MET expression and activation appear to be important for initiation and progression of triple-negative breast cancer. Tivantinib (ARQ 197) is an orally administered agent that targets MET, although recent preclinical data suggests the agent may have mechanisms of action that are independent of MET signaling. We conducted a phase 2 study of tivantinib monotherapy in patients with metastatic triple-negative breast cancer. *Methods* Patients with metastatic triple-negative breast cancer who had received 1 to 3 prior lines of chemotherapy in the metastatic setting were enrolled into this two-stage, single arm phase 2 study. Treatment consisted of twice daily oral dosing of tivantinib (360 mg po bid) during a 21-day cycle. Patients underwent restaging scans at 6 weeks, and then every 9 weeks. Tumor biomarkers that might predict response to tivantinib were explored. *Results* 22 patients were enrolled. The overall response rate was 5 % (95 % CI 0–25 %) and the 6-month progression-free survival (PFS) was 5 % (95 % CI 0–25 %), with one patient achieving a partial response (PR). Toxicity was minimal with only 5 grade ≥3 adverse events (one grade 3 anemia, one grade 3 fatigue, and 3 patients with grade 3/4 neutropenia). *Conclusion* This study represents the first evaluation of tivantinib for the treatment of metastatic triple-negative breast cancer. These results suggest that single agent tivantinib is well tolerated, but did not meet prespecified statistical targets for efficacy.

## Introduction

Breast cancer is the most commonly diagnosed cancer and the second leading cause of cancer death in American women [1]. Despite recent advances in breast cancer treatment, metastatic disease remains incurable. One possible limitation of current therapies has been an inability to select subsets of patients most likely to benefit from specific agents. With the application of gene expression array technology, the heterogeneity of breast cancer has become clearer and the identification of novel cancer subtypes has reinvigorated the search for more specific and effective treatments. Hierarchical clustering of genomic expression data from breast cancer specimens has demonstrated several distinct tumor subgroups with unique expression profiles, including a HER2-positive subgroup, two estrogen-receptor (ER) driven groups, and a “basal-like” group [2]. The basal-like tumors have a poor prognosis relative to other subtypes, even with the best available therapy. Approximately 10–15 % of individuals with breast cancer have basal-like disease [3]. While not all basal-like cancers are triple-negative breast cancers, the majority of triple-negative breast cancers have a basal-like phenotype. There are currently no U.S. Food and Drug Administration (FDA)-approved agents for specific management of triple-negative breast cancer, and there is significant need to develop rational therapy for this subtype of the disease.

MET is a membrane receptor normally expressed on cells of epithelial origin. Hepatocyte growth factor (HGF) is the only known ligand of the MET receptor. Upon HGF stimulation, MET induces several biologic responses that lead to invasive growth. MET overexpression, with or without gene amplification, has been reported in a variety of malignancies, including breast, colorectal, lung, gastric, and hepatocellular carcinoma [4, 5]. Elevated expression of MET has been associated with poor prognosis in breast cancer [6, 7]. Interestingly, data suggest that hepatocyte growth factor (HGF), the ligand for MET, and MET are expressed to a greater degree in triple-negative breast cancer relative to other breast cancer subtypes [8–11]. Mouse models also suggest a critical role for the MET pathway during the development of triple-negative breast cancer. Mice harboring an activating mutant MET knock-in or mutant MET transgene under mouse mammary tumor virus promoter, develop breast cancers with a triple-negative phenotype [12, 13]. Additionally, a MET-driven pathway gene expression signature clustered with basal and triple-negative breast cancer from human tissue samples and correlated with worse patient outcome [12, 13]. These studies suggest that MET expression and activation are important for initiation and progression of triple-negative breast cancer.

Early preclinical work suggested that tivantinib (ARQ 197) binds to and inhibits MET in vitro, and phase I and II trials in a variety of malignancies demonstrated the safety and tolerability of tivantinib, and suggested promising activity of this agent [14–17]. Because MET expression is increased in the triple-negative subset of breast cancer and is associated with worse prognosis, targeting MET signaling in this population is a rational therapeutic strategy. We therefore conducted a single-arm phase 2 study of tivantinib monotherapy in unselected patients with metastatic triple-negative breast cancer.

## Patients and methods

### Patient eligibility

Patients ≥18 years of age with measurable metastatic triple-negative breast cancer were eligible. Triple-negative status was defined as estrogen receptor negative (<10 % staining by immunohistochemistry (IHC)), progesterone receptor negative (<10 % staining by IHC), and HER2-negative (0 or 1+ by IHC, or FISH <2.0). Patients may have received 1 to 3 prior chemotherapeutic regimens for metastatic breast cancer, and must have been off treatment for 14 days prior to initiation of study treatment. Patients must have recovered from acute toxicities of their prior treatment. Patients may have received prior radiation therapy in either the metastatic or early-stage setting, but radiation was required to be completed at least 14 days prior to initiation of study treatment. Patients were required to have an Eastern Cooperative Oncology Group (ECOG) performance status ≤2, and a projected life expectancy greater than 6 months. They were also required to provide formalin-fixed, paraffin-embedded (FFPE) tumor tissue. Key exclusion criteria included: prior receipt of a MET inhibitor; known brain metastases that are untreated, symptomatic, or require therapy to control symptoms; and QTc > 470 ms. Research was approved by local human research protections programs and institutional review boards, and studies were conducted in accordance with the Declaration of Helsinki.

### Study design and treatment

This was a single-arm, two-stage phase 2 study assessing the efficacy of tivantinib monotherapy in patients with metastatic triple-negative breast cancer. Tivantinib was provided by Daiichi Sankyo/ArQule through the Cooperative Research and Development Agreement (CRADA) program. Treatment consisted of oral dosing of tivantinib at 360 mg twice daily over a 21-day cycle. Patients underwent radiographic restaging at 6 weeks, and then every 9 weeks. Patients with complete or partial RECIST responses continued to receive study treatment, while those with progressive disease were taken off study. Dose reductions for toxicity occurred if patients experienced grade 3 or 4 neutropenia or non-hematologic adverse events. From the starting dose of 360 mg, doses were reduced as needed to 240 mg twice daily, 120 mg twice daily, and 120 mg once daily.

The primary objective of this study was to evaluate the activity of tivantinib, as defined by 6-month progression-free survival (PFS) in patients with metastatic triple-negative breast cancer. The 6-month progression-free survival (PFS) was defined as the proportion of patients still receiving treatment at 6 months, who were alive and free of progression at this time point. Secondary objectives were to evaluate objective response based on RECIST 1.1 criteria, to evaluate MET and phospho-MET expression in archival tumor tissue, and to evaluate the incidence of MET positive circulating tumor cells (CTCs) at baseline.

### Assessment of MET amplification by fluorescence in situ hybridization (FISH) in tissue

MET FISH probe labeled with SpectrumRed and CEP7 reference probe labeled with SpectrumGreen were purchased from Abbott Molecular (Des Plaines, IL). FISH was performed following standard protocols. Briefly, 5 μm tissue slides were baked overnight at 60 °C, deparaffinized, treated in 1 % sodium borohydride for 4 h and heated in pressure cooker for 20 min in citrate buffer (pH = 6). After treatment with 150 μg/ml solution of Proteinase K, slides were fixed in 1 % neutral-buffered formalin and denatured in 70 % formamide for 4 min at 72 °C. Probe was denatured for 5 min at 80 °C and incubated for 30 min at 37 °C for pre-annealing. Hybridization was carried out overnight at 37 °C; post-hybridization slide washes were carried out for 20 min in 50 % formamide/2xSSC at 45 °C, followed by 5 min wash in 1 × SSC at 45 °C. FISH signal evaluation and acquisition were performed manually using filter sets and software developed by Applied Spectral Imaging (Carlsbad, CA). Several fields with at least 50 tumor cells total were captured and ratio of MET to CEP7 signal numbers was calculated. Assessment of ploidy status was done by visual screening of all tumor area; cells with maximum number of signals were recorded. MET amplification was defined as a MET/CEP7 ratio ≥ 2. Samples having a MET/CEP7 ratio from 1.5 and up to 2 were defined as having relative MET gain. Samples with a MET/CEP7 ratio of 1 but with more than two copies of each probe were defined as having polysomy of chromosome 7.

### Assessment of MET amplification in circulating tumor cells

CTCs were enriched from 7.5 mL of a patient’s whole blood at the Circulating Tumor Cell Core Facility (Brigham and Women’s Hospital, Boston, MA) using the Circulating Tumor Cell Profile Kit (Veridex/Janssen Diagnostics, Raritan, NJ). Processed samples were received as cells suspended in 900 uL of buffer. An equal volume of PBS was added before tubes were spun down at 200 × g for 8 min. Supernatant was carefully removed, leaving approximately 60 uL of buffer. Cell pellets were gently resuspended and the suspension was applied on the labeled slide and allowed to dry in the vacuum desiccators at room temperature. Slides were placed in methanol at −20 °C for aging and storage.

For FISH, dried slides were treated in 2 × SSC at 37 °C for 30 min, followed by 10 min of treatment with 0.002 % pepsin solution in 0.01 M HCl at 37 °C and 15 min of fixation in 1 % formalin at room temperature. Slides were dehydrated in the series of ethanols, dried and co-denatured with MET/CEP7 FISH probe (Kreatech/Leica Microsystems Inc., Buffalo Grove, IL) on an 80 °C plate for 2 min. Hybridization was carried out at 37 °C overnight, followed by a 0.4xSSC/0.3 % Igepal wash at 72 °C for 3 min and a 2 × SSC/0.1 % Igepal wash at room temperature for 1 min. Slides were dehydrated in the series of ethanols, and dried before application of Vectashield mounting medium with DAPI (Vector Laboratories Inc., Burlingame, CA). FISH signal evaluation and acquisition were performed manually using filter sets and software developed by Applied Spectral Imaging (Carlsbad, CA).

### Immunohistochemistry biomarker assays

Available archival tumor tissue was tested centrally for MET and phospho-MET protein expression. Four-micrometer-thick sections were baked at 37 °C overnight, then deparaffinized and rehydrated. Endogenous peroxidase activity was blocked with 3 % hydrogen peroxide in methanol. For heat induced epitope retrieval, slides were placed in 10 mmol/L citrate buffer at a pH of 6.0 (Target Retrieval Solution, S1699, DAKO) and then pressure cooked (Biocare Medical, Concord, CA) at 122 °C to between 14 and 17 psi with the cycle lasting, on average, 45 min and a cool-down period of approximately 20 min. Immunostains were performed on an automated instrument (DAKO Autostainer Plus, DAKO). A range of titers was tested for both antibodies, and titers were calibrated using positive control staining. Primary antibodies to MET, clone SP44 (dilution 1:100; Spring Bioscience), phospo-MET, clone Y1234/1235 (dilution 1:50; Cell Signaling) were incubated for 40 min at room temperature. A DAKO polymer secondary antibody system was used (EnVision + System-HRP; DAKO, Carpinteria, CA) and incubated for 30 min in a humid chamber at room temperature. Sections were developed using 3,3-diaminobenzidine (Sigma Chemical, St Louis, MO) as substrate and counterstained with Mayer hematoxylin. External positive controls were also run.

MET and phospho-MET protein expression levels were recorded as percentages of positively stained tumor cells in each of the five intensity categories denoted as zero (no staining), 1+ (weak but detectable), 2+ (mildly distinct), 3+ (moderately distinct) and 4+ (strong). For each tumor, a value was derived by summing up the percentages of cells staining at each intensity, multiplied by the weighted intensity of staining: H-Score = (0 × % at 0) + (1× % at 1+) + (2× % at 2+) + (3× % at 3+). This score produces a continuous variable that ranges from 0 to 300. A positive result was set to an H-Score of 1, defined as a minimum of 1 % of tumor cells staining weakly 1+ [18].

### Whole exome sequencing protocol

In the patient who achieved a RECIST response, tumor and matched germline tissue underwent DNA extraction and whole exome sequencing, using a previously described protocol [19]. After whole exome sequencing, somatic point mutations were identified using MuTect [20]. A heuristic algorithm was then applied to identify somatic variants that may have clinical or biological relevance for the selected patient samples.

## Statistical methods

This study used a Simon optimal two-stage design to control type I error at 10 % and have at least 90 % power to detect the acceptable response rate. The sample size was calculated to distinguish between a response rate of 10 versus 31 %. In the study design, 13 participants were to be enrolled in the first stage. If there were at least 2 participants who were alive and progression free at 6 months, the study would enroll another 13 participants. If there were 4 or fewer of the 26 who were alive and progression-free at 6 months, the regimen would be declared inactive. If there were 5 or more who were alive and progression-free at 6 months, the regimen would be declared active. PFS and 95 % confidence intervals (CIs) are described using Kaplan-Meier methods. The overall response is tabulated. All correlative analyses are exploratory.

## Results

The study was activated in April 2012. Based on slow accrual and external findings suggesting that tivantinib may not actually inhibit MET [21], accrual to the study closed in July 2013 after a total of 22 patients were enrolled.

### Patient characteristics

The baseline characteristics of the patients enrolled into the study are listed in Table 1. The mean age was 55 and patients had received a median of 2 prior lines of chemotherapy for metastatic disease. Most patients had widespread metastatic disease, with 73 % of participants having at least 3 metastatic sites, the most common of which were regional lymph nodes (14 of 22 patients), ipsilateral skin of breast/chest wall (11 of 22 patients), bone (10 of 22 patients) and lung (10 of 22 patients).

**Table 1:** Baseline patient characteristics

Characteristic	Number	Percent
Age		
Mean (SD)	55 (10)	–
Median (Range)	56 (33–73)	–
Race		
White	20	91
Black or African American	2	9
Number of metastatic sites		
1	3	14
2	3	14
≥ 3	16	72
ECOG performance status		
0	16	73
1	6	27
Prior lines of chemotherapy for metastatic breast cancer		
1	8	36
2	10	46
3	4	18

### Response to therapy

**Fig. 1:**
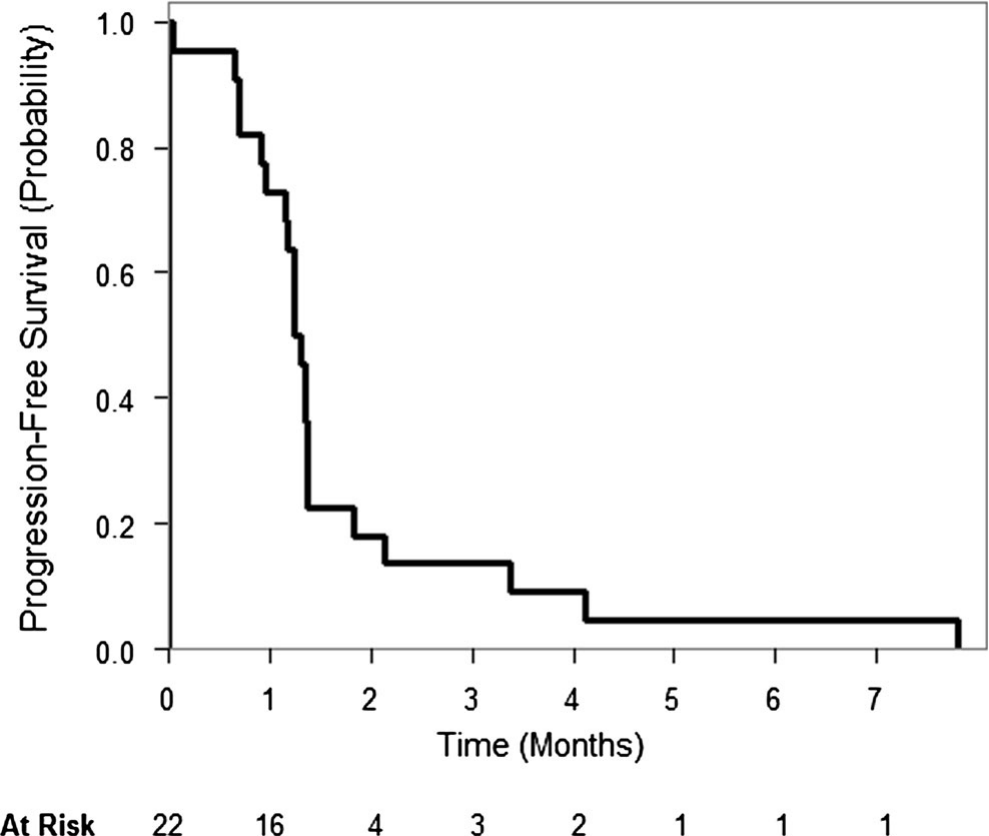
Progression-free survival curves depicting final response to therapy

In all patients, the median progression-free survival (PFS) was 1.2 months (95 % CI: 1.0–1.4), Fig. 1. Only one patient had PFS > 6 months (4.5 %, 95 % CI: 0.2–24.7 %). This patient had disease involving her chest wall, lymph nodes, and pleura. She had a progression free interval of 7.8 months, and progression occurred at a regional lymph node. This patient also achieved a partial reponse (PR). Although the study did not meet the prespecified threshold for continuation, the observation of one partial responder with a PFS > 6 months led the study team to continue accrual to the second stage to further characterize the activity. The overall response rate was 4.5 % (95 % CI 0.1–22.8 %) (Table 2). There were 6 patients (27.3 %) with stable disease as the best overall response by RECIST 1.1. Based on external findings about the mechanism of action of tivantinib [21], and the fact that only 1 patient achieved a PFS > 6 months, accrual to the study closed in July 2013 after a total of 22 patients were enrolled.

**Table 2:** Response rates to tivantinib therapy

Best overall response	Number	Percent
Partial response	1	4.5
Stable disease	6	27.3
Progressive disease	15	68.2

### Toxicity

Few severe adverse events were reported by patients taking tivantinib (Table 3). Of the 46 adverse events reported, 41 were of grade 1 or 2. The most common adverse event was fatigue. There were only 5 grade ≥3 adverse events (one grade 3 anemia, one grade 3 fatigue, and 3 patients experienced grade 3/4 neutropenia).

**Table 3:** Maximum grade reported for agent specific adverse events

Adverse events	Grade 1	Grade 2	Grade 3	Grade 4	Total # of patients (%)
Fatigue	10	5	1	0	16 (73 %)
Nausea	7	0	0	0	7 (32 %)
Anemia	2	3	1	0	6 (27 %)
Diarrhea	4	2	0	0	6 (27 %)
Neutropenia	2	0	2	1	5 (23 %)
Anorexia	2	2	0	0	4 (18 %)
Rash maculo-papular	3	0	0	0	3 (14 %)
Vomiting	2	0	0	0	2 (9 %)

Four dose reductions occurred in 2 patients due to neutropenia and fatigue. Three of four dose reductions occurred within the first two cycles. The one responder required two dose reductions, one in cycle 2 (neutropenia) and another in cycle 8 (neutropenia and fatigue).

### MET expression studies

**Fig. 2:**
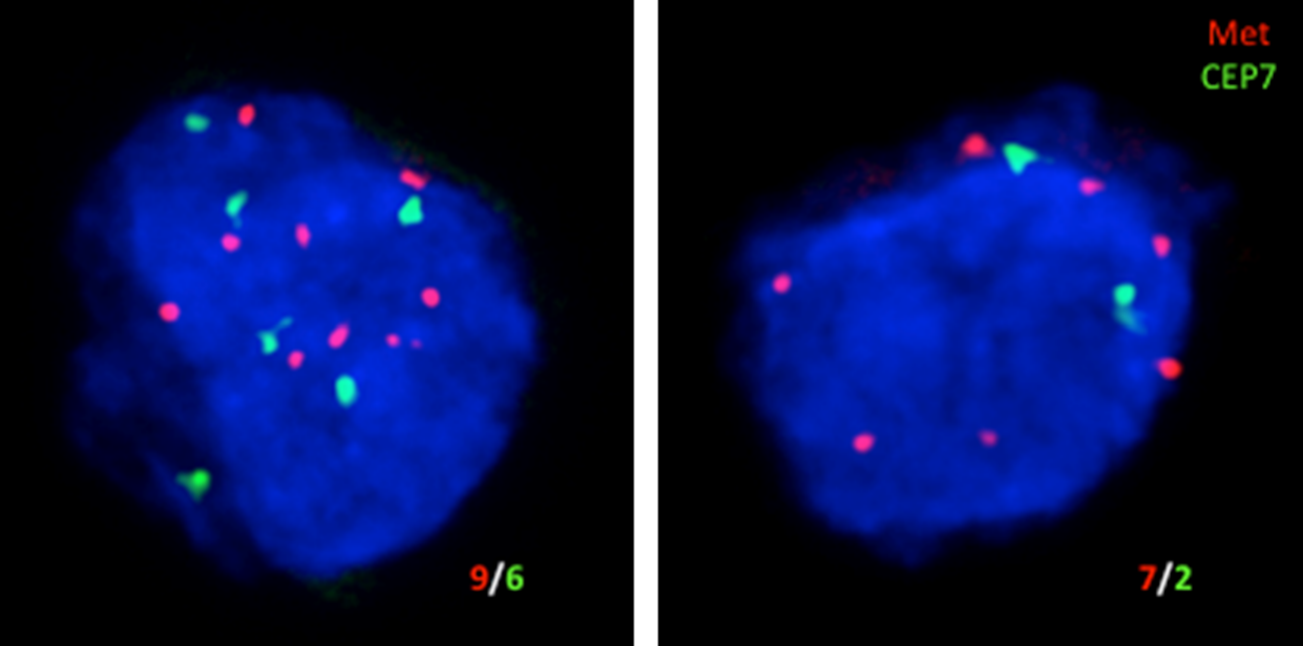
FISH analysis of MET gene on archival tissue of patient with partial response. Most of the 100–120 cells analyzed had ploidy level up to 4n, and the two (~2 % of cells) illustrated below had MET copy number increase (1.5-fold gain and 3.5-fold amplification, respectively)

All 22 enrolled patients had adequate amounts of archival tissue for immunohistochemical (IHC) staining. Among those 22 patients, 10 had positive IHC staining for MET (H-score: median 5, range 1–35), while none of the patients had positive phospho-MET staining on archival tumor tissue. Of 7 patients with circulating tumor cells (CTCs) collected at baseline, 1 had 2-fold MET amplification in her CTCs as assessed by FISH, but this was not correlated with response, as the patient had progressive disease at the first 6 week restaging. Of 18 patients who had FISH testing for MET on archival tissue, 3 were amplified in <5 % of nuclei. Four patients did not have adequate amounts of archival tissue for FISH analysis. The one responder was negative for MET and phospho-MET by IHC on archival tissue, and had CTCs that were not MET-amplified. Her archival tumor tissue was amplified for MET by FISH in less than 2 % of nuclei, though the clinical significance of this is unclear (Fig. 2).

### Whole exome sequencing

Whole exome sequencing of archival tumor tissue and tissue from time of progression in the patient who experienced a PR was attempted. The mean target coverage of the progresion tumor sample was 117 ×. In this sample, somatic mutations in known breast cancer genes MAP2K4 (H79R) and TP53 (R110P) were identified, as well as a variant of unknown significance in the kinase domain of JAK2 (E577Q). No alterations that suggest a mechanism of response or resistance to this agent were found. Unfortunately, this patient’s baseline tumor was unevaluable using this sequencing technology, so it was not possible to determine whether any changes in mutational status led to development of tivantinib resistance.

## Discussion

This trial represents the first study of tivantinib for the treatment of metastatic triple-negative breast cancer. Of 22 patients that were evaluated, only one patient had an objective response. The single responder did not have evidence of MET expression by IHC or clear evidence of MET amplification in her archival tumor specimen, so it is unclear if her response was due to inhibition of MET. To further understand the molecular rationale for the one responder to tivantinib in our trial, we performed whole exome sequencing on tissue from her post-progression biopsy. This analysis identified mutations in known breast cancer genes MAP2K4 and TP53, as well as a mutation in the kinase domain of JAK2. However, the patient’s archival tumor tissue sample was unevaluable, so we could not compare the mutational profile of tivantinib-sensitive versus resistant tissue.

One potential explanation for the low response rate seen in this study was that the population was not enriched for patients with tumors that were high in MET overexpression. Prospective data from studies of tivantinib in the treatment of hepatocellular carcinoma found that positive MET expression (≥2+ staining in ≥50 % of tumor cells by IHC) was associated with improved overall survival (OS), PFS, and time to progression [22]. Additionally, in the MARQUEE trial for patients with advanced non-small cell lung cancer, an exploratory analysis found that patients with tumor that were at least 2+ positive for MET by IHC in more than 50 % of tumor cell experienced longer PFS and OS with combined MET-EGFR inhibition while in patients with MET-low tumors, tivantinib did not improve OS compared to placebo [23]. These data suggest that benefit from tivantinib may potentially be restricted largely to patients with significant MET overexpression, and none of the patients in our study had MET overexpressed in greater than 50 % of the tumor. This may be because MET expression diminishes over time after slides have been cut from paraffin-embedded blocks [24]. For all our patients, samples were analyzed in batch at the end of the clinical trial. Therefore false negativity of samples tested in this study cannot be excluded. Alternatively, it may also be that this level of MET overexpression is uncommon in TNBC. In a randomized phase 2 study evaluating onartuzumab, a monoclonal antibody directed against MET, only 12 % of patients with TNBC had moderate to high expression of MET (IHC 2+/3+ in 50 % of stained tumor cells) [25]. Interestingly, the addition of onartuzumab to either paclitaxel and bevacizumab or paclitaxel alone did not improve PFS or OS compared with paclitaxel and bevacizumab in patients with metastatic or locally recurrent TNBC; additionally, the efficacy in the MET-positive and MET-negative subgroups was similar.

The low response rate in this study may also be related to the possibility that tivantinib may not be a specific inhibitor of MET. Pre-clinical work has revealed that tivantinib binds to MET in vitro, in vivo, and demonstrated disease regression in phase I, II, and III clinical trials [14–17]. However, while the current study was ongoing, several preclinical studies were published suggesting that tivantinib’s mechanism of action may be via anti-tubulin cytotoxic activity. Basilico et al. [26], Katayama et al. [27], and Calles et al. [28] compared the pharmacological profile of tivantinib with that of other selective MET inhibitors in a large panel of human tumor cell lines. They found that tivantinib indiscriminately caused cell death on all actively mitotic tumor cells, regardless of MET gene copy number and MET protein expression. Tivantinib also induced apoptosis in both MET-negative tumor cells and genetically engineered cancer cells expressing a MET protein lacking the tivantinib-binding domain, suggesting that tivantinib acts independently of its ability to bind MET [26]. More recently, data suggests that cytoskeletal and apoptotic changes in cells treated with tivantinib may be mediated by paxilin, a cytoskeleton regulator controlled by MET [3].

While the reason for the low response rate seen in this study is not clear, our results indicate that though tivantinib is well tolerated, it does not have significant clinical efficacy when used as monotherapy to treat unselected, metastatic triple-negative breast cancer.
